# Noise Sources and Requirements for Confocal Raman Spectrometers in Biosensor Applications

**DOI:** 10.3390/s21155067

**Published:** 2021-07-27

**Authors:** Izabella J. Jahn, Alexej Grjasnow, Henry John, Karina Weber, Jürgen Popp, Walter Hauswald

**Affiliations:** 1 Leibniz Institute of Photonic Technology (Leibniz-IPHT), a Member of the Leibniz Research Alliance Leibniz Health Technology, Albert-Einstein-Straße 9, 07745 Jena, Germany; izabella.jahn@leibniz-ipht.de (I.J.J.); alexej.grjasnow@leibniz-ipht.de (A.G.); henry.john@leibniz-ipht.de (H.J.); karina.weber@leibniz-ipht.de (K.W.); juergen.popp@leibniz-ipht.de (J.P.); 2 InfectoGnostics Research Campus Jena, Centre for Applied Research, Philosophenweg 7, 07743 Jena, Germany; 3 Institute of Physical Chemistry and Abbe Center of Photonics, Friedrich Schiller University Jena, Helmholtzweg 4, 07743 Jena, Germany

**Keywords:** confocal Raman spectrometer, biosensor, signal-to-noise, bacteria, fluorescence background

## Abstract

Raman spectroscopy probes the biochemical composition of samples in a non-destructive, non-invasive and label-free fashion yielding specific information on a molecular level. Nevertheless, the Raman effect is very weak. The detection of all inelastically scattered photons with highest efficiency is therefore crucial as well as the identification of all noise sources present in the system. Here we provide a study for performance comparison and assessment of different spectrometers for confocal Raman spectroscopy in biosensor applications. A low-cost, home-built Raman spectrometer with a complementary metal-oxide-semiconductor (CMOS) camera, a middle price-class mini charge-coupled device (CCD) Raman spectrometer and a laboratory grade confocal Raman system with a deeply cooled CCD detector are compared. It is often overlooked that the sample itself is the most important “optical” component in a Raman spectrometer and its properties contribute most significantly to the signal-to-noise ratio. For this purpose, different representative samples: a crystalline silicon wafer, a polypropylene sample and *E. coli* bacteria were measured under similar conditions using the three confocal Raman spectrometers. We show that biosensor applications do not in every case profit from the most expensive equipment. Finally, a small Raman database of three different bacteria species is set up with the middle price-class mini CCD Raman spectrometer in order to demonstrate the potential of a compact setup for pathogen discrimination.

## 1. Introduction

Analytical methods that allow for a non-invasive, label-free analysis of biological samples show multiple advantages over traditional, i.e., immunohistochemistry or immunoassay, techniques [1]. In recent decades Raman spectroscopy has found multiple applications as a biosensor. For example, in histopathology tumor margins were reliably identified [2,3,4], in microbiology pathogen identification can be done on a single cell level [5,6,7] and in pharmacology drug delivery and uptake studies have been performed [8,9]. The Raman effect was discovered in 1928 [10]. Nevertheless, the interest of chemists and biologists was first captured with the advent of laser technology. Raman spectroscopy probes the biochemical composition of the investigated sample in a non-destructive, non-invasive and label-free fashion yielding specific information on a molecular level. It relies on the weak process of inelastic light scattering, where only one out of one million incident photons are inelastically scattered [11]. Therefore, the detection of Raman scattered photons is more challenging, as compared with i.e., fluorescence emission. 

In practice, there are multiple ways to improve the Raman signal by employing: (1) high intensity, monochromatic laser sources, (2) long spectral acquisition times, (3) objectives with high numerical aperture (NA), (4) high-throughput microscopes/spectrometers, (5) detectors with low noise characteristic and/or (6) other variants of Raman spectroscopy. However, the incident laser power on the sample surface has to be carefully adjusted in order to avoid sample damage. A long acquisition time will not only increase the number of detected inelastically scattered photons but also any other photons coming from i.e., fluorescence emission of sample impurities. While high NA objectives offer efficient collection, uneven sample surfaces will lead to the risk of running out of focus and the sampling volume is also significantly decreased. Finally, high-throughput Raman microscopes and spectrometers are the key for obtaining high-quality Raman spectra. Due to the low quantum yield of the Raman effect, reducing the losses within the Raman spectrometer is essential. In principle, different light spectrometer classes (dispersive, filter based, Fourier transform) are available. For Raman spectroscopy in the wavelength window of silicon matrix detectors, dispersive spectrometers are the instruments of choice due to their efficiency and noise performance [12,13]. However, the use of a prism or grating-based spectrometer implies a reduced field-of-view due to the necessary entrance slit. This and thermal considerations suggest a point or slit shaped laser excitation leading automatically to a confocal Raman spectrometer or an instrument/sensor being close to confocal. A remaining crucial parameter is the required spectral resolution. On one hand, a too low spectral resolution will not allow the detection of small spectral differences of similar substances. On the other hand, the full width at half maximum (FWHM) of the Raman bands is influenced by various homogeneous and inhomogeneous broadening processes due to light matter interactions. Increasing the spectral resolution above a certain threshold brings no benefits but additional pixel read noise degrading the signal quality. Typically, Raman devices feature spectral resolutions in the range of 4 cm^−1^ to 10 cm^−1^ [14,15]. In addition to choosing the most suitable Raman instrument, the intensity of the Raman signal can be also significantly improved by employing other Raman spectroscopy techniques. In the last decade many advances on the application of resonance Raman spectroscopy, tip and surface enhanced Raman spectroscopy (TERS, SERS), of coherent anti-Stokes Raman spectroscopy (CARS) or stimulated Raman spectroscopy (SRS) have been published [16,17]. However, all these techniques require either advanced equipment, the use of metallic nanostructures or they can be applied just for specific samples.

Generally, a Raman spectroscopist considers only the intensity of the Raman signal and rarely looks at the combination of signal and noise. The signal-to-noise ratio (SNR) is a good figure of merit to compare the performance of different Raman spectrometers. Sensitive detectors with low readout noise and dark current shot noise are preferred for Raman applications. While the first noise source is independent of the experimental conditions for a given camera setting, the dark current shows both an acquisition time and temperature dependence. Long measurement times and high operating temperatures will lead to significant dark current shot noise levels. Laboratory grade instruments are mostly equipped with deeply cooled charge-coupled device (CCD) detectors in order to reduce this noise source. However, in the last decade, a multitude of mini Raman spectrometers dedicated for a specific application proved to offer good performances. 

In this manuscript we provide a study for performance comparison and assessment of different spectrometers for confocal Raman spectroscopy in biosensor applications. Here in focus: a low-cost, home-build Raman spectrometer with a complementary metal-oxide-semiconductor (CMOS) camera, a middle price-class mini CCD Raman spectrometer and a laboratory grade confocal Raman system with a deeply cooled CCD detector. A discussion regarding how to track back the number of Raman scattered photons on the sample from the number of detected photoelectrons at the detector, as well as, the origin of noise sources influencing the quality of the recorded Raman spectra will be included. These discussions are based on experimental data, as well as on theoretical considerations using information of datasheets from the different components of the Raman setups. As samples, a crystalline silicon (c-Si) wafer, a polypropylene (PP) sample and *E. coli* bacteria are investigated. Therefore, we present three different scenarios: (1) a non-transparent planar sample with high Raman scattering cross-section, (2) a transparent bulk sample and (3) a sample that, in addition to Raman scattered photons, generates a significant fluorescence background. Based on the results we show that biosensor applications do not in every case profit from most expensive equipment. This finding can significantly reduce the size and cost for future Raman sensors in bio-applications. Finally, after consideration of all parameters, a small Raman database of three different bacteria species is set up with the middle price-class mini CCD Raman spectrometer in order to demonstrate the potential of a compact setup for pathogen discrimination.

## 2. Materials and Methods

### 2.1. Instrumentation

Within the framework of this study, the performance of three confocal Raman spectrometers is assessed:

1. a low-cost, small home-build spectrometer with CMOS camera (mini CMOS Raman spectrometer);
2. a middle price-class small Czerny-Turner spectrometer with CCD detector (mini CCD Raman spectrometer);
3. a commercial, laboratory grade, confocal Raman spectrometer with a deeply cooled CCD detector (reference Raman spectrometer).

**Scheme 1:**
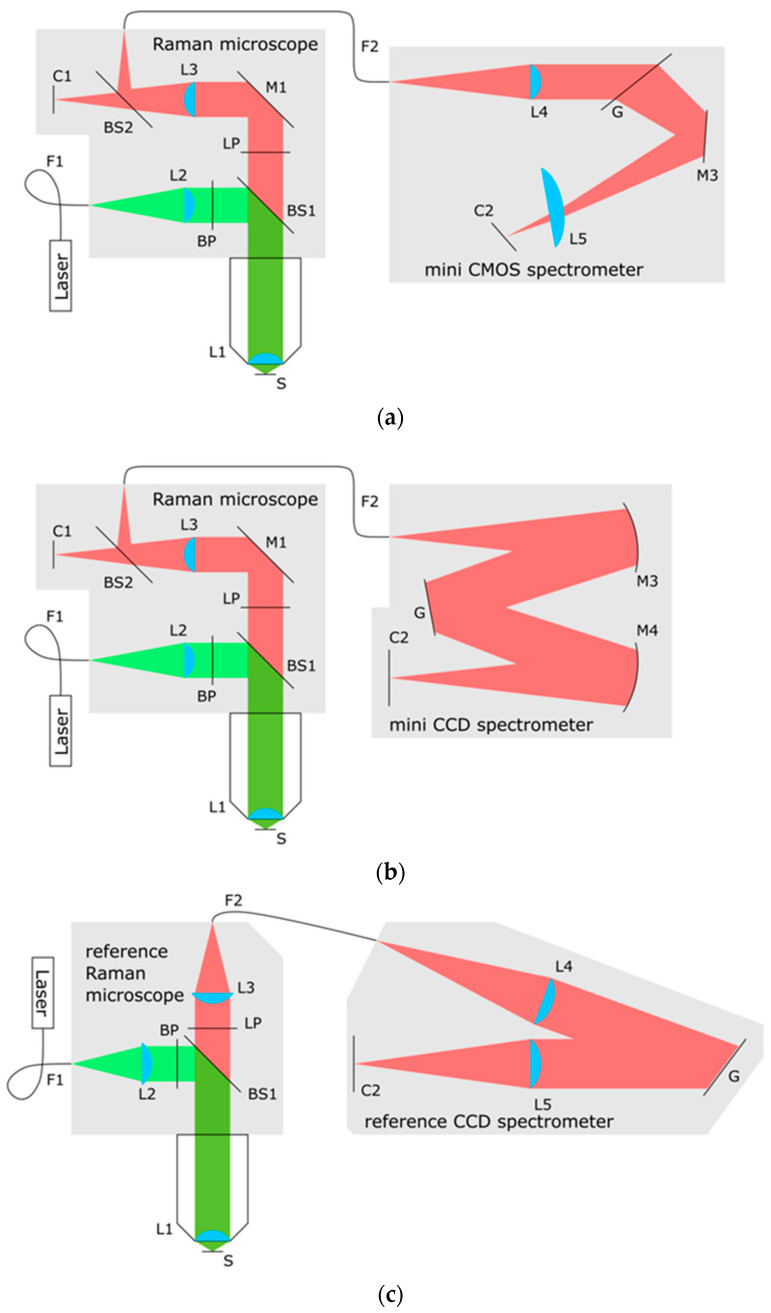
Schematic optical diagram of: (**a**) the mini CMOS Raman spectrometer consisting of Raman microscope, mini CMOS spectrometer and 532 nm laser, (**b**) the mini CCD Raman spectrometer using the same microscope components and laser as (a) but a mini CCD spectrometer, (**c**) the reference Raman spectrometer.

The first two systems are based on the same Raman microscope components and on the same single mode fiber coupled laser (Hübner-Photonics, Cobolt Samba 04-01 Series 532 nm, Kassel, Germany), but feature different fiber (Thorlabs, step-index l = 2 m, d = 25 µm, 0.10 NA, Newton, MA, USA) coupled spectrometers (Scheme 1a,b). For a fair comparison the same microscope objective lens (Olympus MPlanFL N, 50×, 0.8 NA, Tokyo, Japan) **L1** and the same laser power (3.8 mW) at the sample plane **S** are employed throughout the measurements. The set laser power yields optimal results for all samples investigated in the current study. Thermal degradation of all pathogen samples was therefore avoided. The efficiency of optical components was determined between 547 nm (520 cm^−1^) and 660 nm (3650 cm^−1^) whenever possible. The experimental determination at 660 nm only does not pose a problem, since the efficiency of most optical components is almost constant in this range.

#### 2.1.1. Raman Microscope Components

The Raman microscope for the mini CMOS and the mini CCD spectrometer was constructed in the workshop of the Leibniz-IPHT (see Scheme 1a,b left hand side). The excitation laser is coupled in via a collimator lens (Edmund optics, plano-convex lens f = 21.0 mm d = 6.0 mm, Barrington, IL, USA) **L2**, passes through a clean-up filter (IDEX Semrock, 532 nm MaxLine^®^ laser clean-up filter, Lake Forest, IL, USA) **BP** and it is reflected by a dichroic beam splitter (IDEX Semrock, 532 nm laser BrightLine^®^ beamsplitter) **BS1**. The backscattered light is transmitted via the same **BS1** and a long pass filter (IDEX Semrock, 532 nm EdgeBasic™ edge filter) **LP**. One highly reflective aluminum mirror (Thorlabs, 1” × 1” UV-enhanced aluminum mirror, Newton, MA, USA) **M1** is used for folding the light beam. An achromatic lens **L3** (Edmund optics, achromatic doublet lens f = 50.0 mm d = 10.0 mm) acts as a tube lens and a detection fiber coupler. Finally, a 90:10 beam splitter (Thorlabs, 90:10 (R:T) UVFS Plate Beamsplitter with 1 mm thickness, Newton, MA, USA) **BS2** is required for the monochrome widefield camera (Ximea, MU9PM-MBRD, 2592 × 1944 pixels, Münster, Germany) **C1**. The widefield illumination is realized by a low-cost orange light-emitting diode (LED). The sample **S** is mounted movable by micrometer screws against the objective **L1**.

#### 2.1.2. The Mini CMOS Spectrometer

The CMOS-based spectrometer with 0.1 NA (lens speed f/5) was specially designed and built at the Leibniz-IPHT as particularly small and it is based on economic components only (see Scheme 1a right hand side). It features a line width of 12.7 cm^−1^ at 517 nm using a d = 25 µm fiber as entrance slit and a pixel size of 3.5 cm^−1^. To achieve this, a planar, transmissive, curved, chirped and blazed grating **G** was used in addition to a thermoelectrically cooled CMOS **C2** sensor (AMS, CMOSIS, CMV2000 monochrome, 2048 × 1088 pixels, Premstaetten, Austria). The 9 × 13 mm^2^ grating is designed to compensate for aberrations of the spectrometers imaging lenses **L4** (Edmund optics, Achromat f = 36 mm d = 9 mm) and **L5** (Edmund optics, Achromat f = 50 mm d = 18 mm) and fabricated in the clean-room of the Leibniz-IPHT. Its diffraction efficiency of 33% is achieved by a three step sawtooth profile. These structures are realized by electron beam lithography at the Leibniz-IPHT but could be potentially molded. According to the datasheet the chosen CMOS sensor features 60% quantum efficiency (QE), 14 e^−^/pixel readout noise and 125 e^−^/pixel/s dark current at 25 °C. The dark current decreases to ~13 e^−^/pixel/s at the selected operating temperature of 5 °C using a readout electronics manufactured by Codion Optics GmbH, Jena, Germany. A self-written software is used for sensor readout and hardware control. The spectral information of the image sensor is vertically weighted software binned by effectively 21 pixels for each spectral channel.

#### 2.1.3. The Mini CCD Spectrometer

The mini CCD spectrometer has a symmetrical Czerny–Turner configuration (see Scheme 1b) with 75 mm focal length and 0.07 NA (Avantes BV, AvaSpec-ULS2048x64TEC-EVO, Apeldoorn, The Netherlands). It features a line width of 12.1 cm^−1^ at 517 nm using a d = 25 µm fiber as entrance slit and a pixel size of 4.3 cm^−1^. It is equipped with a grating **G** with 1200 grooves/mm (blaze for 750 nm) and a thermoelectrically cooled back-thinned CCD image sensor **C2** with 2048 × 64 pixels (Hamamatsu, S11850-1106, Hamamatsu City, Japan) which is fully vertically hardware binned. According to the datasheet, the sensor features 77% QE, 6 e^−^ readout noise and 2 e^−^/pixel/s dark current at the selected operating temperature of −5 °C. The spectral acquisition was controlled via the AvaSpec 8 software.

#### 2.1.4. The Reference Raman Spectrometer

The laboratory grade confocal Raman micro-spectrometer (Witec, alpha 300R, Ulm, Germany) consists of an upright microscope body (see Scheme 1c) with tube lens (Zeiss, f = 165 mm ICC, Jena, Germany) **L3**, a fiber coupled, continuous-wave, single-frequency laser (Hübner-Photonics, Cobolt, Fandango 150 515 nm, Kassel, Germany) and a fiber coupled spectrometer (Witec, UHTS300 VIS, Ulm, Germany). The laser fiber **F1** is polarization maintaining, single mode with 0.11 NA and the spectrometer fiber **F2** is multi-mode with 50 µm diameter and 0.12 NA. As long pass filter **LP** an (IDEX Semrock, RazorEdge Long Pass U-Grade 514 nm) is used.

The spectrometer with 0.125 NA (lens speed: f/4) consists of two imaging lenses **L4/L5** (Nikon, AF Nikkor 300 mm 1:4 D, Minato, Japan) a grating **G** with 600 grooves/mm and a thermoelectrically cooled (−59 °C) CCD Camera **C2** (Oxford Instruments, Andor iDus DV401A-BV-352, Tubney Woods, UK). It features a line width of 10.3 cm^−1^ at 517 nm using a d = 50 µm fiber as entrance slit and a pixel size of 4.9 cm^−1^. The camera parameters were set as follows: vertical shift speed: 16.25 µs, horizontal shift speed: 33 kHz, preamplifier gain: 1, and the readout mode: single track, 20 rows (from 52 to 72). The spectral acquisition was controlled via Witec Control 4 software or alternatively via Andor Solis software. A widefield camera (not shown in Scheme 1c) is switchable alternatively to the spectrometer, so no extra beam splitter losses occur. The sample **S** is mounted movable by a motorized stage against the objective **L1**.

### 2.2. Bacterial Strains and Culturing Conditions

For testing the signal performance of the Raman spectrometers, the *Escherichia coli* (*E. coli*) strain DSM 423 was used. For the construction of a reference database three independent batches of *E. coli* ATCC 35218, *Pseudomonas aeruginosa* (*P. aeruginosa*) DSM 50071 and *Staphylococcus aureus* (*S. aureus*) ATCC 23213 were measured as well. In order to validate the classification model two other *E. coli* strains (PS1, PS2: isolates from patients) were cultured. All strains were provided by the Institute of Medical Microbiology, University of Jena, and (apart from the patient isolates) were originally purchased from the German Collection of Microorganisms and Cell Cultures (DSMZ) or the American Type Culture Collection (ATCC). All bacterial strains were grown using lysogeny broth (Carl Roth GmbH & Co. KG, Karlsruhe, Germany) agar plates at 37 °C. Prior to the measurement the bacterial cells were harvested from the plates and transferred into a reaction vial with sterile distilled water. This was followed by three washing steps: each time the suspension with the bacterial cells was centrifuged (3000 rcf, 3 min) and the liquid phase was discarded. Then sterile water was added again to the cell pellet and the sample was thoroughly mixed. Finally, the cells were suspended in distilled water and stored at 4 °C until the measurements were performed. For Raman measurements, 3 µL of the suspension were dropped on an aluminum substrate and dried at ambient conditions before measurements. For each Raman spectrometer a new droplet of the bacteria suspension was deposited on the sample carrier. The aluminum substrates used here were previously shown to be suitable for the Raman spectroscopic analysis of bacteria [18]. However, any other chemically and physically stable, biocompatible sample carrier that does not feature interfering Raman bands could be used [19].

### 2.3. Raman Measuremnts

For all measurements the same microscope objective lens (Olympus MPlanFL N, 50x 0.8 NA, Tokyo, Japan) **L1** and the same laser power (3.8 mW) incident on the sample surface was applied. In the case of the standard samples (the polished c-Si wafer with <001> normal orientation and PP), 100 single spectra per measurement position with an acquisition time of 1 s were recorded. The spectra of *E. coli* DSM 423 used for the performance comparison were recorded with an acquisition time of 15 s and 15 single spectra per measurement position. With each device 15 randomly distributed positions on the sample surface were selected. For the Raman database, 25 Raman spectra were recorded for each batch of the bacteria species with an acquisition time of 15 s and 15 acquisitions per measurement position.

### 2.4. Signal-to-Noise Ratio (SNR) Considerations

The experimental SNR for a particular measurement and a selected Raman band is defined as the ratio of the peak intensity S, usually above the baseline, and the standard deviation of the peak intensity N [20]. Furthermore, it is possible to calculate the SNR from a combination of individual noise sources and experimental data, as shown in the following equation:

(1) $$\mathrm{SNR}=\frac{S}{N}=\frac{S}{\sqrt{N_{P}^{2}+N_{b}^{2}+N_{d}^{2}+N_{r}^{2}}}=\frac{S}{\sqrt{S+B+D+N_{r}^{2}}}$$

The signal S can be determined experimentally in units of photoelectrons. For this the knowledge of the conversion factor between analog digital converter units (ADUs) and number of photoelectrons (not photons) is necessary. The signal maximum of the spectrometer is given by the so-called full well capacity of a single pixel (in case of CCD hardware binning sometimes of a dedicated shift register). The cameras amplifier gain is typically chosen to match full well capacity and analog digital converter range.

The noise N can be calculated as a combination of instrumental and experimental sources. Since the noise sources can be regarded as statistically independent, the total variance $N^{2}$ is the sum of the variances $N_{x}^{2}$ of the individual sources:

$N_{p}^{2}$ variance of signal photon noise (shot noise of photons),
$N_{b}^{2}$ variance of background noise (shot noise of fluorescence),
$N_{d}^{2}$ variance of dark current noise (shot noise of dark current) and
$N_{r}^{2}$ variance of readout noise.

Noise due to wrong background estimation and modulation noise of multimode collection fibers is not yet considered. For a detailed discussion of the individual sources (see Section S1 of the Supplementary Information).

### 2.5. Data Pre-Processing and Chemometrical Analysis

Raman spectra of the c-Si, PP and *E. coli* DSM 423 were minimally pre-processed in order not to introduce any artefacts. Data analysis was carried out using self-written scripts in the programming language R [21]. All recorded spectra are dark current corrected. For this, dark current spectra were recorded with the same integration time as the spectra of the samples. The resulting spectra were cut to the 150–3600 cm^−1^ wavenumber region. In order to calculate the experimental SNR the intensity of a selected Raman band was determined for each single spectrum. 

Data included in the Raman database for pathogen identification were pre-processed and analyzed with the software Ramanmetrix^TM^ (Biophotonics Diagnostics GmbH, Jena, Germany) [22,23]. The reference database was built on the Raman spectra acquired from the three batches of *E. coli* ATCC 35218, *S. aureus* ATCC 2913 and *P. aeruginosa* DSM 50071. The Raman spectra of the two *E. coli* strains isolated from patients (PS1 and PS2) were treated as independent data and used to test the discrimination power of the classification model. In the first step all data were subjected to the same pre-processing pipeline. Namely, the spectra were despiked [24], wavenumber calibrated using 4-acetoaminophenol as standard [25], cut to the 400–3100 cm^−1^ wavenumber range, baseline corrected with the sensitive nonlinear iterative peak (SNIP) algorithm [26] and vector normalized. The preprocessed data were subjected to principal component analysis (PCA). For further analysis, the first six principal components (PCs) were considered. The reference database was used to train the classification model based on linear discriminant analysis (LDA). As cross-validation, the leave one batch-out cross validation (LOBOCV) was used. This is a robust method to estimate the performance of supervised models [27]. To predict each batch, a model is constructed on data subsets excluding that batch. This process is repeated until all data are predicted. Finally, the test dataset is fed to the created classification model. 

## 3. Results and Discussion

### 3.1. Photon Budget of Instruments

Comparing the performance of different confocal Raman spectrometers is not trivial. The Raman intensity values, often expressed in ADUs, obtained from the instrument software should not be directly compared in absolute values. These numbers are related just with the number of photoelectrons read out by the detector and not with the actual generated number of Raman scattered photons at the sample plane. Any loss or optical aberration within the Raman spectrometer will lead to a lower number of detected photoelectrons. The first step toward assessing the signal quality of the three confocal Raman spectrometers is to convert the measured spectra in ADUs to units of absolute photoelectron counts. For this, the conversion factor of the camera has to be known/calculated. The converted values can then be compared with the values calculated for a physically ideal micro-spectrometer. This allows us to reveal the presence of coupling losses and to evaluate the significance of each noise source.

To calculate the conversion factor from camera ADUs into absolute photoelectron counts at the detector, the well-established mean-variance plot method [28] is used for this research (see details in the Supplementary Information, Section S2 with Figures S1 and S2). This method makes use of the known Poisson statistics of photons. It works very well when being applied to raw data, but runs the risk of misinterpretation for algorithmically pre-treated data. The latter happens in the time series measurement mode of the reference system when using the WITec Control 4 software but can be avoided using the Andor Solis software for the same system. The experimentally determined conversion factors are very close to the values listed in the data sheets. This confirms the robustness of the mean-variance plot method (see Table 1).

For comparison with a physically ideal Raman micro-spectrometer the expected absolute photon number at a c-Si sample using the optical phonon mode at 519 cm^−1^ is calculated using experimental values determined from the measured spectra (Table S1) as well as parameters from the literature. Unfortunately, some of the necessary optical reference parameters of c-Si like the Raman scattering cross section and the imaginary refractive index are determined with poor accuracy. Furthermore, the actual emission geometry of the sample is angle and polarization dependent for high NA optics. Moreover, multiple references for the Raman scattering cross section exist [29,30]. As an upper estimate of the achievable signal, a simplified emission model (scalar, homogeneous and isotropic) is applied (see Section S3 of the Supporting Information) and the higher reference cross section value is chosen.

With the knowledge about measured photoelectrons and theoretically generated and detectable photons at the sample, a theoretical Raman spectrometer detection efficiency can be calculated (see Table 1). These values are surprisingly low, so that a discussion of the factors that make up this loss is necessary. The relative optical efficiency of the three Raman spectrometers is however assessable already.

In order to explain the theoretical efficiency, the values are split into optical losses explainable by single optical components in use (optical efficiency—see Table 1 and Supplementary Information for details) and a remaining efficiency. The remaining efficiency (see Table 1 and Table S2) includes all unrevealed losses, coupling losses (caused by aberrations, vignetting, and pollution) and a potential gain overestimation of the ideal micro-spectrometer.

Table S2 shows that most of the optical losses of the reference Raman spectrometer might be correctly identified (1.146 × 10^6^ photons/s practically vs. (4.2 ± 2.7) × 10^6^ photons/s ideally). Comparing both mini Raman spectrometers with the reference (13% and 19% vs. 27%) shows additional loss which can be attributed to aberrations and undiscovered coupling losses in the Raman microscope components used for both systems and within the home-build mini CMOS spectrometer.

In order to perform Raman measurements with the shortest possible exposure time and the lowest possible illumination, the total collection efficiency of the optics should be as high as possible. The detailed analysis of three very different confocal Raman spectrometers shows where an optimization is worthwhile and where it is only possible with high effort. However, a high signal is only a necessary, but not a sufficient, criterion for information-rich Raman measurements. Therefore, the known absolute signal is put in relation to the noise sources, leading to the discussion of SNR in the next section.

### 3.2. Signal-to-Noise Ratio of Spectra

A high SNR leads to low detection limits in quantitative analysis and improved performance of e.g., statistical methods for pathogen discrimination. Therefore, comparing the SNR of different spectrometers gives valuable information on the performance and limitations of the Raman setup. It is often overlooked that the sample is the most important “optical” component in a Raman spectrometer and its properties contribute most significantly to the SNR. For this comparison, different representative samples: c-Si, PP and a film of *E. coli* bacteria were measured under similar conditions using the three confocal Raman spectrometers described above.

#### 3.2.1. Samples

The first sample is an opaque planar c-Si wafer. This type of sample is commonly used for Raman instrument adjustment due to its high Raman scattering cross-section and sharp band located around 519 cm^−1^. The Raman scattered photons are expected to be generated in a single plane of the sample. Thus, small differences in the confocal volume of different instruments do not have a major impact on the recorded spectrum.

In the case of the second sample, a transparent PP piece, the laser light can penetrate deeper, as compared with the case of c-Si, in the sample and the Raman scattered photons are collected from a 3D volume. Here, a microscope with a larger confocal volume will collect more inelastically scattered photons. Therefore, a signal gain relative to the case of a planar c-Si wafer is expected for the microscope with larger confocal volume.

Finally, as in biosensor applications, the researchers often have to deal with samples showing significant fluorescence background. A dried suspension of *E. coli* bacteria was also investigated and the performance of the mini CMOS and mini CCD spectrometers are compared with the reference CCD spectrometer.

#### 3.2.2. Raman Spectra

**Figure 1:**
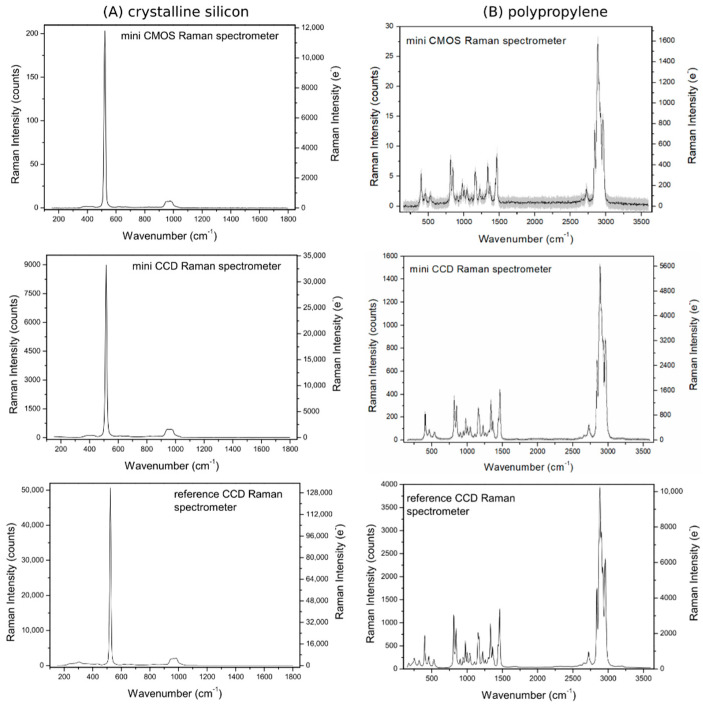
Dark current corrected mean Raman spectra of (**A**) crystalline silicon wafer and (**B**) polypropylene measured with the three platforms (1 s acquisition time, 100 single spectra). For better comparison the Raman intensity is expressed both in counts and e^−^.

**Figure 2:**
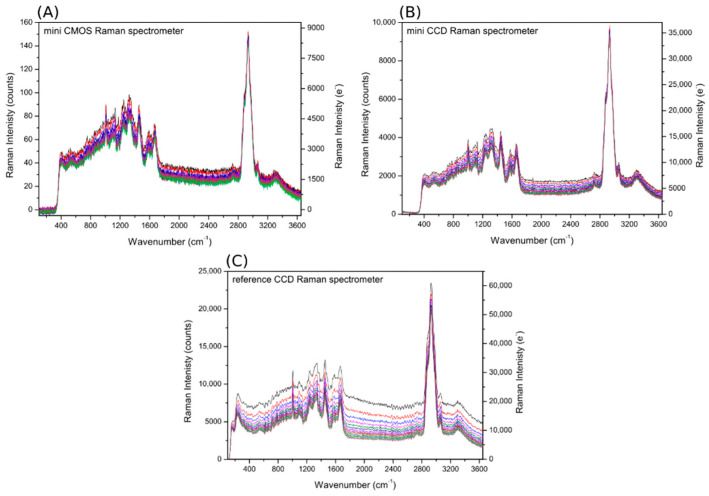
Raman spectra of *E. coli* DSM 423 bacteria measured with (**A**) the mini CMOS Raman spectrometer, (**B**) the mini CCD Raman spectrometer and (**C**) the reference CCD Raman spectrometer at a single position on the surface of the aluminum substrate. The spectrum plotted in black is the first spectrum in the series, while the green one is the last spectrum.

The Raman spectra of c-Si and PP recorded with the three Raman spectrometers are presented in Figure 1, while the ones of the dried bacteria are shown in Figure 2. For the c-Si sample the Raman mode centered around 519 cm^−1^, assigned to the first-order longitudinal optical phonon mode, was chosen as a marker band. The intensity of this band in units of photoelectron counts for the three Raman spectrometers is listed in Table 2. 

At first look, all spectrometers deliver similar spectra. In Figure S3 only the spectral region between 490 and 550 cm^−1^ is shown. For better comparison, the spectra were horizontally shifted and the marker Raman band was fitted with a Voigt function. The FWHM ranges from 12.7 to 10.3 cm^−1^. The mini CMOS spectrometer shows the widest band with the highest pixel resolution. The differences come from the different gratings and dispersion optics used; nevertheless, they are comparable for all three spectrometers. 

PP has a smaller Raman scattering cross section as c-Si. This can be also noticed by comparing the Raman intensities of the two samples for a given instrument (Figure 1 and Table 2). As the Raman marker band, the most intense peak at 2888 cm^−1^ assigned to the symmetrical CH_2_ stretching vibration, was selected for discussion.

In the case of many biological samples an autofluorescence background is present in the recorded Raman spectra and can significantly reduce the SNR to a level where the Raman bands are not detectable anymore. In order to simulate such a scenario a dried suspension of *E. coli* DSM 423 bacteria on an aluminum substrate was investigated. During the experiments 15 randomly distributed positions on the sample surface were selected and measured. At each position 15 spectra were recorded with 15 s exposure time per spectrum. For each spectrometer the measurement position with the best spectra in terms of intensity and signal stability was chosen and considered for further analysis. The dark current corrected single Raman spectra at a given position are plotted in Figure 2. In these results it is obvious that a photo bleaching of the autofluorescence background takes place, with stronger effect being noticed for the reference CCD spectrometer. This is caused by the smaller confocal volume of this Raman spectrometer. Furthermore, the fluorescence intensity of the first spectrum in the series is higher for the reference CCD Raman spectrometer. Before each measurement, a quick check of the Raman signal was performed with a 1 s spectral acquisition time by slightly moving the sample stage on the z axis to achieve the best signal. Due to the fast and well aligned wide-field camera of the reference CCD Raman spectrometer, the sample could be placed in an optimal focus already in the bright-field mode. Consequently, the time required for finding the best focal plane was shorter for this setup leading to reduced photo-bleaching. For a fair comparison only the last spectrum of the measurement series is considered, assuming that a maximum photo bleaching level was achieved. 

The recorded spectra of the *E. coli* show Raman bands characteristic for this type of samples. For example, around 1000 cm^−1^ a sharp Raman band assigned to the ring breathing vibration of phenylalanine is observed, as well as the high intensity band at 2932 cm^−1^ assigned to CH_2_ vibrations of proteins. The later Raman band is selected for the discussion on SNR and its intensity for the three Raman spectrometers is listed in Table 2.

#### 3.2.3. The Role of Confocality

The mini CMOS and mini CCD Raman spectrometers have approximately two times larger confocal volume as compared to the reference CCD Raman spectrometer. This means, in the case of a transparent bulk sample (PP, *E. coli*) vs. an opaque planar sample (c-Si), the first two spectrometers will collect more Raman scattered photons as compared with the reference CCD Raman spectrometer. This can be clearly seen by normalizing the signal intensity of the marker Raman bands. As standard, the signal intensity of the given sample determined from the spectrum recorded with the reference CCD spectrometer is used. The values are listed in Table 2 (normalized signal intensity) and support the presented hypothesis. Namely, for PP the normalized signal intensity in case of both mini spectrometers is two times higher as compared with the case of c-Si. A larger confocal volume is also advantageous for collecting Raman spectra from dried bacteria suspensions. Here, even a larger relative signal is observed (Table 2). This might be caused by the high reflectivity of the aluminum surface used as sample substrate for the bacteria. 

Considering that the SNR is calculated as the ratio of mean signal intensity and noise sum, a larger confocal volume will have a positive impact on the calculated and experimental SNR in case of transparent bulk samples.

#### 3.2.4. Raman Signal Independent Noise Sources

The readout noise is the only noise source independent of the experimental conditions. In the current study, it was determined using the data provided by the producer of the detectors. For a detailed calculation the reader is referred to Table S3 in the Supporting Information. There exists a significant difference between the CMOS and the CCD technology. In the case of the mini CMOS spectrometer, the spectral information of the image sensor is vertically weighted software binned (about 21 pixels), while in the case of the mini and reference CCD spectrometers the spectral information is hardware binned and readout only once. Therefore, the total readout noise for the mini CMOS spectrometer is significantly larger, 54.73 e^−^/px (RMS), as compared with the two other spectrometers, 6 and 7 e^−^/px (RMS), respectively (Table S3). 

The dark noise depends on both: the acquisition time and the sensor temperature. Efficient cooling significantly reduces the dark noise. The reference Raman spectrometer operates at −59 °C, and therefore it is characterized by a low dark current (0.03 e^−^/px/s). Although the CCD camera has 127 pixel rows, only the rows (20) containing spectral information are read out, further reducing the total dark noise per spectral channel. In the case of the mini CCD spectrometer all pixel rows are binned. This combined with an operating temperature of −5 °C leads to an increased dark current level, in comparison with the reference CCD spectrometer. Finally, the mini CMOS spectrometer is characterized by the highest dark current level (Table S3).

In Table 2 the sum of the two Raman signal independent noise sources is listed. In the case of c-Si and PP the same spectral acquisition time of 1 s was used, while for the bacteria 15 s were used. Longer acquisition times lead to higher dark current shot noise and consequently to larger sum noise values. Note, in case of the bacteria fluorescence shot noise is considered as a third Raman signal independent noise source.

#### 3.2.5. Calculated vs. Experimental SNR—The Case of Non-Fluorescing Samples

The two standard samples, c-Si and PP, when excited with a laser emitting at 515 nm or 532 nm do not present any luminescence/fluorescence background in the recorded Raman spectra. Therefore, for the calculation of the SNR only the signal shot noise, dark current shot noise and the readout noise, but no background noise, have to be considered in Equation (1). Based on Table 2, in the case of c-Si at 1 s spectral acquisition time, the signal shot noise dominates the dark shot and the readout noise for all spectrometers. The reference CCD spectrometer provides the highest SNR. In the case of PP, the signal intensity for a given spectrometer at the set acquisition time is lower as compared with the c-Si sample due to the smaller Raman scattering cross section. For the two CCD spectrometers the signal shot noise limit is still valid, however in the case of the mini CMOS spectrometer the dark current shot noise and the readout noise have a significant influence on the SNR at 1 s spectral acquisition time. 

In Table 2 the values of the experimental SNR for c-Si and PP are also listed. These were calculated using the 100 single spectra recorded with the three spectrometers. While, for the mini CMOS and mini CCD spectrometer the experimental and the calculated SNR values are very similar, in the case of the reference CCD spectrometer considerably higher experimental SNR were obtained. This could mean that either the information obtained from the datasheet of the CCD camera producer are erroneous, or the experimental data are better than what theory predicts, or there is some data handling process hidden within the spectral acquisition software mend to improve the results. The first two options do not seem plausible; therefore, the third scenario was investigated in detail. Theoretically, no two spectra, measured even with perfectly identical experimental conditions, are identical due to the random nature of light matter interaction. In order to test if this is true for the recorded spectra the correlation matrix for each dataset was computed. The results are represented as color maps and are included in the Supporting Information, Figure S4. Each pixel of the map displays the correlation between two spectra. The main diagonal shows that each spectrum always perfectly correlates with itself and it corresponds to a correlation factor equal with 1. In the maps computed for the mini CMOS and mini CCD spectrometer noise correlations are shown only by the main diagonal. For the reference CCD spectrometer also the second diagonal displays a very high correlation that is close to 1. This means that the spectra are not independent and probably an averaging step is performed during the readout of the CCD using Witec Control 4 software leading to improved SNR beyond shot noise limit.

#### 3.2.6. Calculated SNR—The Case of Fluorescing Samples

The Raman spectra of *E. coli* DSM 423 bacteria plotted in Figure 2 present a strong fluorescence background. Therefore, in Equation (1), in addition to the noise sources present for the two standard samples discussed above, the fluorescence shot noise also must be considered. The fluorescence intensity was measured at 1800 cm^−1^ in the silent region of the Raman spectrum (Figure S5). In the case of the reference CCD spectrometer the fluorescence shot noise is significant, 86.07 RMS e^−^/px, as compared with the sum of dark current shot noise and readout noise, 7.62 RMS e^−^/px (see Section S5 in the Supplementary Information) but still lower as the signal shot noise. The amount of fluorescence photons detected by the camera is independent of the detector characteristics. Therefore, cooling to low temperatures cannot reduce the fluorescence shot noise. This observation is further supported by the data presented in Table 2. Namely, the performance of the mini and of the reference CCD spectrometer is very similar. Of note, the mini CCD Raman spectrometer features about twice the confocal volume than the reference. As already discussed above, this will allow for the collection of more Raman scattered photons in the case of transparent bulk samples. The performance of the mini CMOS spectrometer is strongly impaired by the high dark current level at long spectral acquisition times. An experimental SNR cannot be determined correctly for all spectrometers due to the presence of the photobleaching process. 

#### 3.2.7. The Influence of Acquisition Time on the SNR

**Figure 3:**
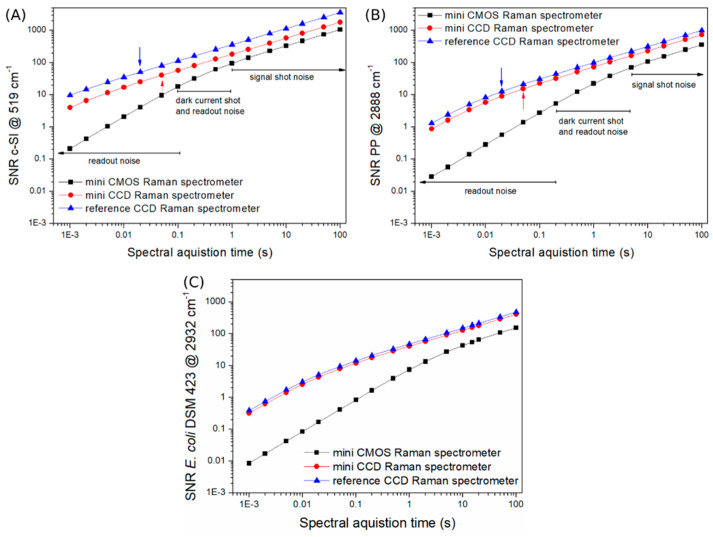
Spectral acquisition time dependent SNR on double logarithmic scale for selected Raman marker bands of (**A**) c-Si, (**B**) PP and (**C**) *E. coli* DSM 423 theoretically calculated for the three spectrometers. In (**A**,**B**) the time domains where different noise sources dominate are indicated: mini CMOS Raman spectrometer with three domains, mini and reference CCD Raman spectrometers with two domains indicated by arrows. In (**C**) four noise sources compete. The delimitation of time domains is complex.

In Figure 3 the change in the SNR as function of the spectral acquisition time is depicted. The representation of the data on double logarithmic scale allows the identification of various time domains where different noise sources dominate. For the standard samples these regions are marked on the graphs (Figure 3A,B). Namely, for the case of the mini CMOS spectrometer at short acquisition times, the readout noise, at intermediate times, the dark current and readout noise, and at long acquisition time the signal shot noise dominates. For the mini CCD and reference CCD spectrometer the readout noise dominates at short integration times, while above the signal shot noise is more significant. The reference CCD spectrometer is cooled to −59 °C, and therefore the dark current shot noise is negligible. In the case of the mini CCD spectrometer, due to the time dependent characteristics of the dark current shot noise, this noise source becomes significant at acquisition times where the signal shot noise dominates.

Figure 3C clearly illustrates that for samples with fluorescence emission a middle price-class CCD spectrometer delivers similar results as a laboratory grade CCD spectrometer. Therefore, for specific applications the overall costs of a Raman platform can be significantly reduced. Due to the contribution of four different noise sources a clear delimitation of different time domains, as for the standard samples, is challenging. For very short acquisition times the readout noise dominates. Due to the considerably higher efficiency of fluorescence emission as compared to Raman scattering, the signal shot noise starts to dominate at much longer acquisition times as compared with the case of the two standard samples. This means, in order to record Raman spectra of these type of samples a longer acquisition time has to be used during the experiments. Comparing the three instrument graphs for a given sample and a desired SNR (in horizontal direction), highlights the difference in required exposure time on all instruments necessary for gaining equal spectral information quality.

#### 3.2.8. Other Noise Sources

In the Raman spectra of the *E. coli* bacteria recorded with the reference CCD spectrometer a signal modulation, ~5%, in the silent region is observed. Due to the low operating temperature the dark current shot noise does not cover this effect. In other experiments (with fluorescent slides), not shown here, the same effect and magnitude was noticed also for the mini CMOS and mini CCD spectrometers. Generally, spectrometers equipped with a multimode fiber for light collection show this multiplicative amplitude modulation due to mode interference which is highly dependent on the exact bending of the fiber and may look just like noise. These modulation changes during the acquisition of large data sets can significantly affect the performance of statistical models applied to the Raman data. This phenomenon can be avoided by: (1) Raman spectrometers without collection fiber or (2) the use of a single mode collection fiber or (3) a very long fiber. The third solution leads to a high delay between modes and therefore to not resolvable spectral high-frequency modulation. In other words: the modes are added quasi incoherently on the detector of the spectrometer.

Overall, the SNR is a good measure to compare the performance of different Raman spectrometers. By carefully considering all noise sources, a clear image on the advantages and limitations of a given setup can be made. From the results presented in this section it is obvious that the choice of Raman spectrometer highly depends on the intended use. For samples without fluorescence background systems similar to the reference CCD Raman spectrometer will provide optimal results. For samples with a fluorescence background, such as many biological samples, CCD detectors without an extraordinary deep cooling can be used. This will significantly reduce the overall costs of the instrumentation. Finally, the economic CMOS technology features high quantum efficiency but comes without suitable hardware binning and is rarely coolable due to modern ball grid array packages. Even though current CMOS sensors achieve a readout noise of less than 3 electrons per pixel, the dark current shot noise, critical for the required long exposure times, is physically limited and makes cooling necessary for any kind of silicon based detector [31]. However, the cooling does not need to reduce the dark current shot noise significantly below the fluorescence shot noise of the sample, avoiding the need of elaborated fogging protection of the sensor in some case.

### 3.3. Pathogen Identification

Raman spectroscopy provides fingerprint specific information about the biochemical composition of the sample, allowing the discrimination between various bacteria species even on the strain level [32,33,34]. Based on the results presented above, the mini CCD Raman spectrometer performs similarly with a laboratory grade Raman spectrometer for the analysis of bacteria samples, with the advantage of reduced instrumentation costs. In order to test the potential of the mini CCD Raman spectrometer to be applied in a clinical setting for pathogen identification a database containing spectra of three different bacteria species has been established. Pathogenic strains of *S. aureus* and *P. aeruginosa* are often associated with the development of infections of the respiratory tract, while *E. coli* is one of the most commonly encountered pathogens in the human body being part of the normal microbiota. Hence, the discrimination between these three species is of clinical interest. 

**Figure 4:**
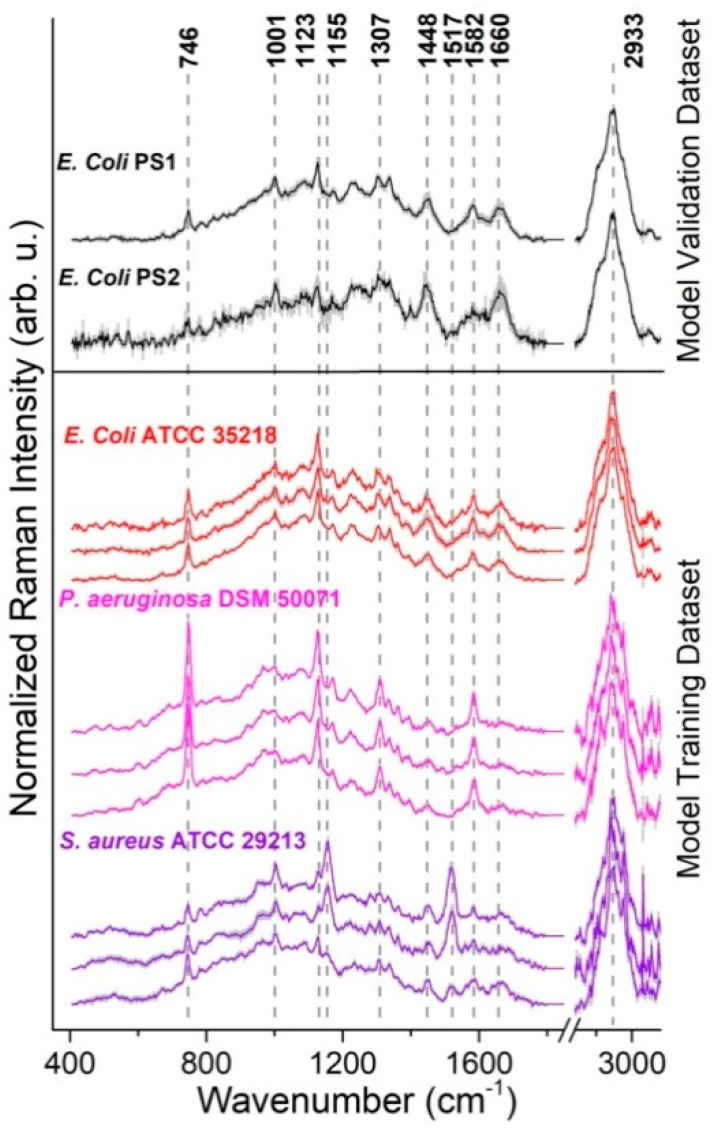
Mean Raman spectra and their double standard deviation of bacteria species included in the Raman database (3 independent batches/bacterium species for the PCA-LDA model training dataset).

For the discrimination of the bacteria species the PCA-LDA supervised statistical method was applied. The reference database was measured with the mini CCD Raman spectrometer and it includes three independent batches for each bacterium species that were cultured and measured on three different days. It is known, that the spectral variations are larger within batches as between bacteria cultured in one batch [35]. Furthermore, Raman spectra are sensitive to changes of the measurement setup. This can negatively influence the results. In Figure 4 the mean Raman spectra and their double standard deviation of the three batches of each bacterium species are shown. All spectra present Raman bands characteristic for the different cell components, such as proteins and lipids (i.e., at 746, 1001, 1448, 1660 and 2933 cm^−1^). In addition to these bands, the Raman spectra of *S. aureus* show strong features centered at 1155 and 1517 cm^−1^. These indicate the presence of carotenoids [36]. In the case of the third batch, the intensity of these Raman modes is lower as compared with the first two batches. This highlights the importance of including not just one batch in the database. The Raman spectrum of *P. aeruginosa* also presents three intense bands (at 1123, 1307 and 1582 cm^−1^) that cannot be attributed to cell components, but can be ascribed to cytochrome C. Taking into account the wavelength of the laser used for the measurements, 532 nm, and the absorption spectrum of carotenoids and cytochrome C the high intensity of these Raman bands can be explained by the occurrence of an electronic absorption in the range of the Raman excitation wavelength and therefore the resonance Raman enhancement condition is met.

For each batch 25 Raman spectra were measured at randomly selected positions. Therefore, for each bacterium species 75 spectra were included in the reference database. As described in a previous section, for building the statistical model PCA was used for dimension reduction followed by LDA analysis. Here the first six PCs were considered. The performance of the PCA-LDA model was assessed by the LOBOCV. The resulting confusion table is shown in Table 3A. In total, 223 spectra out of 225 were correctly classified resulting in an accuracy of 99.1%. The two *S. aureus* spectra that were as *E. coli* classified had a low signal-to-noise ratio. 

To confirm the predictive capacity of the classification model two other *E. coli* bacteria strains isolated from patient samples at the Institute of Medical Microbiology, University of Jena, were cultured and spectra were measured. The before-built PCA-LDA model was used to predict this independent test dataset to check for overfitting. As presented in Table 3B all 55 spectra were correctly classified as *E. coli*, leading to an accuracy of 100%. 

## 4. Conclusions

Comparing the performance of different confocal Raman spectrometers is not trivial but important in order to be able to choose the most suitable instrumentation for the intended application. In this study the performance of three confocal Raman spectrometers was assessed and compared. The mini CMOS and mini CCD Raman spectrometer, feature the same Raman microscope but different spectrometers: one in the lower price range and one in the middle price range. The third confocal Raman spectrometer is a laboratory grade Raman system with a deeply cooled CCD detector.

One way to assess the performance of a Raman spectrometer is to compare it with a physically ideal micro-spectrometer. This allows to reveal the presence of coupling losses and to evaluate the significance of each noise source. Here we showed that while for the reference CCD Raman spectrometer most losses could be correctly identified, for the other two Raman spectrometers losses attributed to aberrations and undiscovered coupling effects must be present.

By using the SNR as a figure of merit we show that the sample itself plays a very important role. Namely, in the case of standard samples, such as c-Si and PP, the reference CCD Raman spectrometer outperforms the two other devices. This is due to the low readout and dark current shot noise of the detector. However, when the sample causes an auto fluorescence background, the fluorescence shot noise plays a decisive role. The middle-price class mini CCD Raman spectrometer proved to have a very similar performance with the reference CCD Raman spectrometer. The reason is that image sensor cooling does not need to reduce the dark current shot noise significantly below the fluorescence shot noise of the sample. This avoids the need of sensor temperatures below the freezing point and so the need of elaborated fogging protection paving the way to potent, economic and portable confocal Raman biosensors. Furthermore, we also show that the economic CMOS camera is strongly impaired by a high noise level and although it is a cost-effective alternative, it can probably be applied only for samples with high Raman scattering cross-section.

A strong recommendation is to not utilize multimode collecting fibers for spectroscopy in case of the presence of a high continuous auto fluorescence background to avoid systematic spurious signatures in the recorded spectra. For the measurements of pure substances with strong decisive spectral features the multiplicative amplitude modulation caused by fiber mode interference might be negligible.

Finally, we prove that the compact low-cost home-built confocal Raman microscope combined with the mini CCD spectrometer can be successfully applied for pathogen identification. The use of the home-built Raman microscope is not limited to the investigation of planar samples. Microfluidic platforms or cuvettes could be also easily integrated on the sample stage giving the possibility of studying a large variety of samples.

## Figures and Tables

**Table 1:** Efficiency comparison between the three different confocal Raman spectrometers and the ideal Raman micro-spectrometer. Note that the product of optical efficiency and remaining efficiency gives the theoretical efficiency. Note also that the data refers to the detection in the entire confocal Raman spectrometer (consisting of microscope, fiber coupling and spectrometer with sensor) and not only to the spectrometer (see Section S4 of the Supplementary Information for discussion).

Raman Spectrometer	Mini CMOS	Mini CCD	Reference	Units
Excitation laser wavelength	532	532	514	nm
Laser power at the sample	3.8	3.8	3.8	mW
Conversion factor (datasheet)	-	4	2	e^−^/ADU
System magnification	13.9	13.9	45.8	x
Collection fiber diameter	25	25	50	µm
Integrated c-Si signal 519 cm^−1^	816	28,065	119,948	ADU/s
Conversion factor measured	58 ^(a)^	3.7	2.6	e^−^/ADU
Photoelectrons at detector	0.047 × 10^6^	0.104 × 10^6^	0.312 × 10^6^	e^−^/s
Photons ideally expected at the sample ^(b)^	(5.2 ± 3.5) × 10^6^	(5.2 ± 3.5) × 10^6^	(4.2 ± 2.7) × 10^6^	1/s
Theoretical efficiency ^(c)^	0.92%	2.01%	7.42%	
Optical efficiency ^(d)^	7.0%	10.4%	27%	
Remaining efficiency ^(e)^	13%	19%	27%	

^(a)^ The mean-variance plot of the utilized maximally amplified CMV2000 gives 2.6 e^−^/ADU/pixel. Additional vertically weighted software binning leads here to a 22.3 factor. ^(b)^ Assuming an ideal Raman micro-spectrometer with 0.8 NA, no photon losses and a homogeneous and isotropic scattering sample (see Section S4 and Table S2 of the Supplementary Information for discussion). ^(c)^ The theoretical efficiency compares between practically generated photoelectrons at the detector and theoretically generated detectable photons at the sample. ^(d)^ The optical efficiency is explainable by the properties of single optical components in use (see Section S4 and Table S2 of the Supplementary Information for discussion). ^(e)^ Comparison between practically and ideally expected photon numbers at the sample: deviations show unrevealed coupling losses (due to aberrations, absorptions and vignetting) and the potential overestimation of the theoretical model.

**Table 2:** Calculation of SNR for c-Si, PP and *E. coli* bacteria related to a single spectral class. For further information see Section S5, Tables S3 and S4 of the Supplementary Information.

Raman Spectrometer		Mini CMOS	Mini CCD	Reference	Units
Signal Intensity c-Si (519 cm^−1^)		11,714	33,253	131,542	e^−^
Normalized signal intensity Si		0.09	0.25	1	ref
Signal free noise sum ^a^ (1 s)		57.23	12.81	7.04	e^−^/px
Signal shot noise		122	183	363	e^−^/px
SNR_c-Si_	Calculated ^b^	96	182	363	
	Experimental ^c^	87	169	505	
Signal Intensity PP (2888 cm^−1^)		1569	5590	10,129	e^−^
Normalized signal intensity PP		0.15	0.55	1	ref
Signal free noise sum ^a^ (1 s)		57.23	12.81	7.04	e^−^/px
Signal shot noise		*40*	*75*	*101*	e^−^/px
SNR_PP_	Calculated ^b^	23	77	100	
	Experimental ^c^	24	71	141	
Signal Intensity (S) *E. coli* (2932 cm^−1^)		6960	29,711	42,185	e^−^
Normalized signal intensity		0.16	0.7	1	ref
Raman signal free noise sum ^a^ (15 s)		95.03	74.54	86.40	e^−^/px
Signal shot noise		*83*	*172*	*205*	e^−^/px
SNR*_E. coli_*	Calculated ^b^	55	158	185	
	Experimental ^c^	N.A. ^d^	N.A. ^d^	N.A. ^d^	

Obs.: ^a^—sum of dark current shot and readout noise, in case of *E. coli* also including fluorescence shot noise; ^b^—ratio (in one spectral class) of measured mean signal intensity and sum noise (including the signal shot noise); ^c^—ratio of measured mean signal intensity and standard deviation of the signal intensity; ^d^—due to photobleaching the value cannot be determined correctly.

**Table 3:** (**A**) PCA-LDA results for classification model and (**B**) identification of an independent test data set measured with the mini CCD spectrometer.

**(A)**					
	**Predicted**			**Sensitivity** **(%)**	**Specificity** **(%)**
**True**	***E. coli***	***P. aeruginosa***	***S. aureus***		
*E. coli*	75	0	0	100	99
*P. aeruginosa*	0	75	0	100	100
*S. aureus*	2	0	73	97.3	100
**(B)**					
	**Predicted**			**Sensitivity** **(%)**	**Sensitivity** **(%)**
**True**	***E. coli***	***P. aeruginosa***	***S. aureus***		
*E. coli*	55	0	0	100	n/a
*P. aeruginosa*	0	0	0	n/a	100
*S. aureus*	0	0	0	n/a	100

## Data Availability

The data presented in this study are available on reasonable request from the corresponding author.

## Supplementary Materials

The following are available online at https://www.mdpi.com/article/10.3390/s21155067/s1, (Section S1) Signal-to-noise ratio (SNR) and individual noise sources, (Section S2) Conversion factor calculation from camera ADUs into absolute photoelectron counts at the light detector: Figure S1 Emission spectrum of a tungsten halogen lamp filtered with a B18 filter and detected by the three Raman spectrometers presented in the current study, Figure S2. Mean-variance plot for conversion factor estimation, (Section S3) Expected absolute number of collectable Raman photons scattered by the optical phonon mode of silicon at 519 cm^−1^ using a confocal microscope, Table S1: Calculating the integrated photon number per second ideally emitted by the Raman mode of silicon at 519 cm^−1^ using a confocal microscope, (Section S4) Detailed photon budget of instruments, Table S2: Detailed efficiency comparison between the three different confocal Raman spectrometers and the ideal Raman micro-spectrometer, (Section S5) Calculation of noise contribution—standard and biological samples, Table S3. Overview of noise sources: standard samples: c-Si and PP, Table S4. Overview of noise sources: biological sample: *E. coli* DSM 423, Figure S3. Raman marker of c-Si sample band fitted with a Voigt function: background corrected and normalized to unity. (A) original and (B) corrected wavenumber axis, Figure S4. Map of correlation matrix, Figure S5. Dark corrected Raman spectrum of *E. coli* DSM 423 measured with the three Raman spectrometers (the last one of the measurement series at a selected position).
