# Supplementary material for: Noise Sources and Requirements for Confocal Raman Spectrometers in Biosensor Applications
Source: Sensors (Basel). 2021 Jul 27;21(15):5067. doi: 10.3390/s21155067 (PMC8348363; doi:10.3390/s21155067)
Supplement: Supplementary file 1 [file sensors-21-05067-s001.zip › sensors-1298284-supplementary.pdf]

Supplementary Information: Noise sources and requirements for confocal Raman spectrometers in biosensor applications

Izabella J. Jahn [1,3], Alexej Grjasnow [1,3], Henry John [1], Karina Weber [1,2,3], Jürgen Popp [1,2,3] and Walter Hauswald [1,3*]

1 Leibniz Institute of Photonic Technology (Leibniz-IPHT), a member of the Leibniz Research Alliance Leibniz Health Technology, Albert-Einstein-Straße 9, 07745 Jena, Germany; izabella.jahn@leibniz-ipht.de, alexej.grjasnow@leibniz-ipht.de, henry.john@leiniz-ipht.de, karina.weber@leibniz-ipht.de, juergen.popp@leibniz-ipht.de
2 Institute of Physical Chemistry and Abbe Center of Photonics, Friedrich Schiller University Jena, Helmholtzweg 4, 07743 Jena, Germany;
3 InfectoGnostics Research Campus Jena, Centre for Applied Research, Philosophenweg 7, 07743 Jena, Germany
* Correspondence: walter.hauswald@leibniz-ipht.de; Tel.: +49 3641 206 313

1.) Signal-to-noise ratio (SNR) and individual noise sources

The SNR is the ratio of a signal S in units of photoelectrons and the standard deviation N of the combined individual instrumental and experimental noise sources, as shown in the following equation:

$$SNR = \frac{S}{N} = \frac{S}{\sqrt{N_P^2 + N_b^2 + N_d^2 + N_r^2}} = \frac{S}{\sqrt{S + B + D + N_r^2}} \quad (1)$$

Since the noise sources can be regarded as statistically independent, the total variance N^2 is the sum of the variances N_x^2 of the individual sources.

N_p^2 variance of signal photon noise (shot noise of Raman signal photons)

The signal photon shot noise N_p arises from the counting process of Raman scattered photons S at a selected spectral position. If this is the only noise source, referred to as the sample shot noise limit, the maximal SNR value is achieved and it is equal with $\sqrt{S}$. An increase of exposure time or of incident laser power on the sample will linearly increase the signal but additionally increases the shot noise by a square root.

N_b^2 variance of background noise (shot noise of fluorescence)

The background B is caused by any detected photon arising from the environment, directly the laser or sample impurities causing autofluorescence [1]. For biological samples, autofluorescence usually has a significant impact on the background noise. Theoretically, with a good estimation of this background B it is possible to remove its expectation value completely by baseline correction. However, the additional photon noise N_b is not removable. An increase of exposure time or of incident laser power will linearly increase the background B except of ambience light, which is of cause independent of the laser power.

N_d^2 variance of dark current noise (shot noise of dark current)

The dark current D arises from thermally generated electrons in the detector, it is strongly temperature dependent and is accompanied by an additional shot noise N_d. By cooling the detector, the dark current noise can be significantly reduced. Experimentally, the expectation value of the dark current can be measured by recording spectra in the absence of light. An increase of exposure time linearly increases the dark current D. Note

that the dark current is not only accumulated while exposure but between sensor flush and readout. So slow or delayed readout does add dark current.

N_r^2 variance of readout noise

Finally, the readout process refers to a shift of generated photoelectrons and a serial analog amplification which produces an extra signal independent noise N_r according to the readout bandwidth. Experimentally it can be determined by recording spectra with short exposure time (where dark current can be neglected) in the absence of light. The readout noise reduces with readout speed. Since readout noise is a camera characteristic, being independent of any experimental parameter, the cameras amplifier gain is typically chosen to match this readout noise to at least two ADUs, so that additional quantization noise of the ADC is negligible in any case. For the typically long exposure times necessary for Raman spectroscopy readout noise becomes negligible if no excessive software binning is applied. For biological samples with high autofluorescence background it might be then worth splitting a long exposure into many shorter ones enabling for photo bleach and carbonization observations.

Additional noise sources

Noise sources which are not considered in equation (1) are post processing noise (for example due to wrong background estimation) and mode interference noise of multi-mode collection fibers. The later manifests itself as a multiplicative fluctuation in spectral response, which depends on the exact bending of the multimode collection fiber. Depending on the fiber profile the modulation depth typically ranges around 3-7%. For identification of pure substances without heavy background, this type of noise is less serious. For biological samples which feature a high background and are chemically almost similar, this additional modulation can easily become the dominant source of noise. It does not occur for single mode fiber spectrometer coupling and in fiber free Raman spectrometers.

2.) Conversion factor calculation from camera ADUs into absolute photoelectron counts at the light detector

The Raman intensity values obtained from the software of a Raman sensor are expressed in analog digital converter units (ADUs). In order to calculate the conversion factor from camera ADUs into absolute photoelectron counts at the detector, the well-established mean-variance plot method can be applied [2]. The Raman scattered photons arrive in an uncorrelated manner at the detector and produce photoelectrons with a certain quantum efficiency. These generated electrons follow the Poisson distribution, where the variance of the photoelectron count it is equal with its mean. The conversion factor or gain is the slope of the line regression variance vs. mean. Note: The conversion factor is independent of the detector´s quantum efficiency.

For the current research, a tungsten halogen lamp was used as a light source. The emitted light is filtered by a long pass filter BG18 (SCHOTT AG) before it is coupled in to the collecting fiber of the spectrometer. The recorded raw spectra are shown in Figure S1. Due to the filter a smooth variation of the intensity is obtained over the spectral window. In Figure S2 the mean-variance plots are represented. The different pixel values are distributed in 100 classes. The mean variance plot and calculation of the conversion factor was performed in Matlab using a self-written algorithm without prior dark current correction. The obtained values are specified in each graph and are in a good agreement with the data provided by the camera producers.

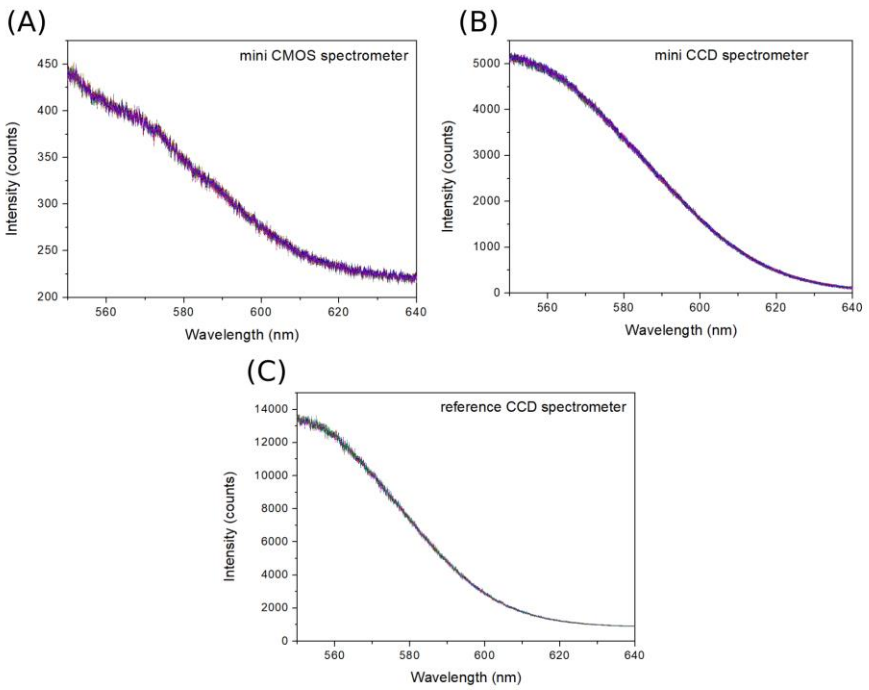

Figure S1. Raw emission spectrum of a tungsten halogen lamp filtered with a B18 filter and detected by the three Raman spectrometers presented in the current study. A time series of 100 spectra with 100 ms acquisition time were recorded with each device.

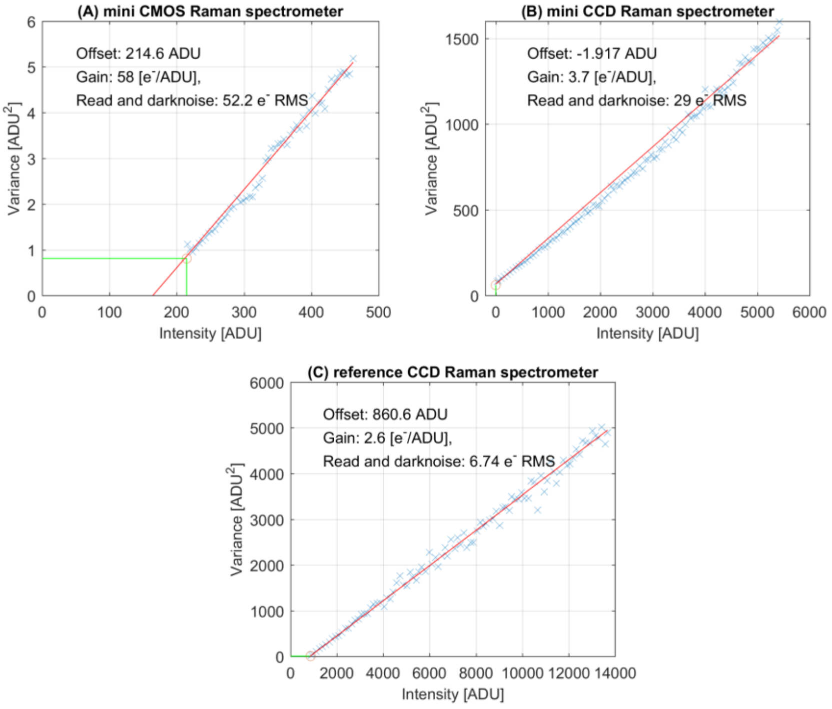

Figure S2. Mean-variance plots for conversion factor estimation of the three Raman spectrometer. A series of 100 dark spectra and 100 spectra of the light emitted by a tungsten halogen lamp and filtered by a bandgap filter (BG 18) were acquired with 100 ms acquisition time. Note: the average read and darknoise of the mini CCD Raman spectrometer is notably higher than the noise of some pixels with low dark current.

3.) Expected absolute number of collectable Raman photons scattered by the optical phonon mode of silicon at 519 cm^{-1} using a confocal Microscope

It is challenging to measure the actual generated number of Raman scattered photons, since any loss or optical aberrations lead to underestimations. In order to absolutely validate the photon budgets of the three Raman spectrometers presented, reference values such as the absolute Raman scattering cross section and the penetration depth of light can be used for calculations. In fact, the reference values required for this are sometimes only determined to an accuracy of 45%. In order to validate at least the order of magnitude, corresponding estimates for silicon (Si) from [3] are adapted here to confocal microscopy.

For our measurements and calculations, we use a polished silicon wafer with <001> normal orientation (parallel to optical axis in z-direction). According to the polarizability tensor χ of Si, an electromagnetic pump field with polarization in <100> direction (x-axis) produces Raman scattered light with polarization in <010> direction (y-axis) and vice versa. Note: due to the high NA objective lenses used for confocal microscopy, polarization mixing occurs. As an upper estimate of the achievable signal, we apply a scalar model assuming a homogeneous and isotropic radiating sample volume.

The Raman collection efficiency is given by:

$$\eta_c = \frac{\Omega}{4\pi \cdot n_{Si}^2(\lambda_{E0})}$$

where Ω is the solid angle of light collected by the 0.8 NA microscopy lens in air:

$$\Omega = 2\pi\left(1 - \sqrt{1 - \left(\frac{NA}{n_0}\right)^2}\right)$$

The maximum possible solid angle of emission outside silicon is 2π but inside silicon the high refractive index of the material at the vacuum emission wavelength $n_{Si}(\lambda_{E0})$ reduces the collection efficiency outside.

The Raman scattering cross section is given by:

$$\sigma_{RS}(\lambda_{P0}) = \frac{F \cdot h\frac{c_0}{\lambda_{P0}}}{\eta_c P_P N L}$$

where F is the integrated photon number per second emitted by the Raman mode (integrated peak area in spectrum around 519 cm^{-1}), $h\frac{c_0}{\lambda_{P0}}$ is the pump Photon energy, P_P is the pump laser power, N is the particle number density of silicon and L is the effective sample thickness which contributes to the scattering. This leads directly to:

$$F = \sigma_{RS}(\lambda_{P0}) \cdot \frac{P_P \cdot N \cdot L \cdot \lambda_{P0}}{h \cdot c} \cdot \frac{\Omega}{4\pi \cdot n_{Si}^2(\lambda_{E0})}$$

For almost transparent samples the contributing sample thickness is limited by the axial resolution of the confocal microscope, which can be calculated roughly as [4]:

$$L = \frac{n\lambda}{2NA^2}$$

where n is the average refractive index of the medium (at pump and emission wavelength) and λ is the average pump and emission wavelength.

Silicon however has a much shorter penetration depth for visible light. The contributing sample thickness is instead limited by the penetration depth of pump and emission light and further reduced by reflexion:

$$L(\lambda_{P0}, \lambda_{E0}) = \frac{\left(1 - R_{Si}(\lambda_{P0})\right)\left(1 - R_{Si}(\lambda_{E0})\right)}{\alpha_{Si}(\lambda_{P0}) + \alpha_{Si}(\lambda_{E0})}$$

with $R_{Si}(\lambda_{P0})$ being the reflectance of silicon at the pump wavelength and $R_{Si}(\lambda_{P0})$ being the reflectance of silicon at the emission wavelength:

$$R_{Si}(\lambda) = \frac{(n_{Si}(\lambda) - 1)^2}{(n_{Si}(\lambda) + 1)^2}$$

and $\alpha_{Si}(\lambda_{P0})$ and $\alpha_{Si}(\lambda_{E0})$ are the absorption coefficients for the pump or the emission wavelength respectively:

$$\alpha_{Si}(\lambda) = \frac{4\pi \cdot k(\lambda)}{\lambda}$$

were $k(\lambda)$ is the imaginary refractive index (absorption) of silicon. Calculations of the photon numbers per second F used in the manuscript are given in Table S1.

Table S1: Calculating the integrated photon number per second ideally emitted by the Raman mode of silicon at 519 cm^{-1} using a confocal microscope with 532 nm laser and 515 nm laser. Intermediate results are printed in italics.

**Physical quantity**	**Symbol**	**for 532 nm**	**for 515 nm**	**Unit**
Pump wavelength	λ_{P0}	532	515	nm
Emission wavelength	λ_{E0}	547	529	nm
Raman scattering cross section [3]	$\sigma_{RS}(\lambda_{P0})$	(4.6 ± 2.0) · 10^{-26}	(5.2 ± 2.3) · 10^{-26}	cm^2
Laser power	P_P	3.8 ± 0.1	3.8 ± 0.1	mW
Particle number density	N	4.992 · 10^{22}	4.992 · 10^{22}	cm^{-3}
Solid angle of collection	Ω	2.513	2.513	
Refractive index at λ_{P0} [5]	$n_{Si}(\lambda_{P0})$	4.140	4.215	
Refractive index at λ_{E0} [5]	$n_{Si}(\lambda_{E0})$	4.089	4.159	
Imaginary refractive index at λ_{P0} [5]	$k_{Si}(\lambda_{P0})$	0.05027 ± 0.01	0.06098 ± 0.01	
Imaginary refractive index at λ_{E0} [5]	$k_{Si}(\lambda_{E0})$	0.04083 ± 0.01	0.05216 ± 0.01	
Effective sample thickness	$L(\lambda_{P0}, \lambda_{E0})$	*186 ± 41*	*142 ± 25*	nm
Reflectance of silicon at λ_{P0}	$R_{Si}(\lambda_{P0})$	*0.373*	*0.380*	
Reflectance of silicon at λ_{E0}	$R_{Si}(\lambda_{E0})$	*0.368*	*0.375*	
Absorption coefficient at λ_{P0}	$\alpha_{Si}(\lambda_{P0})$	*0.00119 ± 0.00024*	*0.00149 ± 0.00024*	nm^{-1}
Absorption coefficient at λ_{E0}	$\alpha_{Si}(\lambda_{E0})$	*0.00094 ± 0.00023*	*0.00124 ± 0.00024*	nm^{-1}
**Photons per second**	**F**	**(5.2 ± 3.5) · 10^6**	**(4.2 ± 2.7) · 10^6**	s^{-1}

Note: The Raman scattering cross section for Si and a 515 nm pump is given in [3] but for 532 nm pump it is calculated assuming a λ^4 law. The measured Raman scattering cross section for Si and a 515 nm pump given in [3] is 1.2 times larger as the calculated one in [6]. The imaginary refractive indexes of silicon are calculated from a fitted linear function from 492 nm to 563 nm using values of [5].

Using a silicon wafer sample is an economic way of calibrating absolute photon numbers of a confocal Raman microscope. Due to the high uncertainties of reference values and the advanced collection geometry in confocal microscopy using high NA optics, the method should only be used to estimate or validate the order of magnitude. For a fast diagnosis of an unknown confocal Raman spectrometer the method helps to reveal a heavy misalignment of the confocal pinhole, aberration issues and unwanted absorption losses.

4.) Detailed photon budget of instruments

In Table S2 a detailed photon budget of both mini and the reference Raman spectrometer, including single component optical efficiencies is given and discussed.

Table S2: Detailed efficiency comparison between the three different confocal Raman spectrometers and the ideal Raman micro-spectrometer. The theoretical efficiency of the three Raman spectrometer is calculated as ratio of measured photoelectrons and ideally collectable photons. In order to explain the low theoretical efficiency values, they are split into the two factors: optically losses explainable by single optical components in use (total optical efficiency) and a remaining efficiency. The remaining efficiency is attributed to additional system and coupling losses and overestimations by the theoretical model.

**Raman spectrometer**		**mini CMOS**	**mini CCD**	**reference**	**units**
Excitation laser wavelength		*532*	*532*	*514*	*nm*
Laser power at the sample		*3.8*	*3.8*	*3.8*	*mW*
Conversion factor datasheet		*not known*	*4*	*2*	*e^-/ADU*
System magnification		*13.9*	*13.9*	*45.8*	*x*
Collection fiber diameter		*25*	*25*	*50*	*µm*
Si signal at 519 cm^{-1}		816	28065	119948	ADU/s
Conversion factor measured		58	3.7	2.6	e^-/ADU
Photoelectrons at detector		$0.047\cdot10^6$	$0.104\cdot10^6$	$0.312\cdot10^6$	e^-/s
Photons ideally expected at the sample		$(5.2 \pm 3.5)\cdot10^6$	$(5.2 \pm 3.5)\cdot10^6$	$(4.2 \pm 2.7)\cdot10^6$	1/s
**Theoretical efficiency**		**0.92%**	**2.01%**	**7.42%**	
component optical efficiency	Sensor a)	60%	77%	97%	
	Sensor oblique a)	88%	unknown	100%	
	Grating b)	33%	65%	70%	
	Spectrometer lens (mirror) c)	95%	unknown	70%	
	Confocal pinhole (ideal) d)	90%	90%	83%	
	Poor fiber NA match	100%	49%	100%	
	Filter e)	92%	92%	92%	
	90:10 beam splitter	90%	90%	100%	
	Microscope f)	76%	76%	100%	
	Objective lens g)	75%	75%	75%	
**Total optical efficiency**		**7.03%**	**10.42%**	**27.22%**	
Photons practically expected at the sample		$0.6728\cdot10^6$	$0.4884\cdot10^6$	$1.1456\cdot10^6$	1/s
**Remaining efficiency h)**		**13.02%**	**19.29%**	**27.26%**	

a) The efficiency is averaged between 547 nm (Si 520 cm^{-1}) and 660 nm (>CH 3650 cm^{-1}) from the datasheets of the sensors and cameras [7-9]

b) Measured in case of mini CMOS at 660nm, estimated in case of mini CCD and from handbook in case of reference [10]

c) Determined from datasheet in case of mini CMOS, estimated from typical photographical lens in case of reference

d) Coupling efficiencies for confocal microscopy [10]

e) Efficiency of dichromatic mirror and long pass filter

f) Additional losses of the microscope (aluminum mirror 83%, lenses and apertures) measured at 660 nm

g) Measured at 660 nm

h) Most of the optical losses of the reference Raman spectrometer might be correctly identified. Comparing both mini Raman spectrometers with the reference shows additional loss which can be attributed to aberrations and undiscovered coupling losses in the Raman microscope components used for both systems and both spectrometers.

5.) Calculation of noise contribution – standard and biological samples

While the experimental SNR was determined only from measured Raman spectra of the samples, for the calculated SNR a combination of experimental data (signal intensity at a selected spectral pixel or pixel column) and data provided by the detector´s producer were taken into account [7-9]. Note that this direct comparison is fair because all three spectrometers feature approximately the same spectral pixel size (See Figure S1).

Table S3. Overview of noise sources: standard samples: c-Si and PP

	Raman spectrometer	mini CMOS	mini CCD	reference CCD	units
Read-out noise	*Read noise*	*14.00*	*6.00*	*7.00*	e^-
	Nr. of reads	*15.28* a)	*1*	*1*	
	Total read noise	54.73	6.00	7.00	RMS e^-/px
Dark current shot noise	*Dark current*	*13*	*2*	*0.03*	e^-/px/s
	Nr. of rows	*21.6*	*64*	*20*	
	Acquisition time	*1*	*1*	*1*	*s*
	Total dark current	*280.80*	*128.00*	*0.60*	e^-/px
	Dark current shot noise	16.76	11.31	0.77	RMS e^-/px
	**Signal free noise sum**	**57.23**	**12.81**	**7.04**	**RMS e^-/px**
	Conversion Factor	*58*	*3.7*	*2.6*	e^-/ADU
Signal shot noise	*Signal Intensity (c-Si)*	*202*	*8987*	*50593*	*counts*
		11714	33253	131542	e^-
	**Signal shot noise (c-Si)**	**122**	**183**	**363**	**e^-/px**
	Signal Intensity (PP)	*27*	*1511*	*3896*	*counts*
		1569	5590	10129	e^-
	**Signal shot noise (PP)**	**40**	**75**	**101**	**e^-/px**

a) The spectral information of the image sensor is vertically (Gaussian weighted) software binned by effectively 21.6 pixels for each spectral channel leading to 15.28 times pixel read noise.

Table S4. Overview of noise sources: biological sample: *E. coli* DSM 423

	Raman spectrometer	mini CMOS	mini CCD	Reference CCD	units
Read-out noise	*Read noise*	*14.00*	*6.00*	*7.00*	e^-
	Nr. of reads	*15.28* a)	*1*	*1*	
	Total read noise	54.73	6.00	7.00	RMS e^-/px
Dark current shot noise	*Dark current*	*13*	*2*	*0.03*	e^-/px/s
	Nr. of rows	*21.6*	*64*	*20*	
	Acquisition time	*15*	*15*	*15*	*s*
	Total dark current	*4212*	*1920*	*9*	e^-/px
	Dark current shot noise	64.90	43.82	3.00	RMS e^-/px
	**Signal free noise sum**	**84.89**	**44.23**	**7.62**	**e^-/px**
Fluorescence	*Fluorescence level*	*31.44*	*973*	*2849*	*counts*
		1823.52	*3600.10*	*7407.40*	e^-
	**Fluorescence shot noise**	**42.70**	**60.00**	**86.07**	**RMS e^-/px**
	Conversion Factor	*58*	*3.7*	*2.6*	e^-/ADU
Signal shot noise	*Signal Intensity*	*120*	*8030*	*16225*	*counts*
		6960	*29711*	*42185*	e^-
	**Signal shot noise**	**83**	**172**	**205**	**e^-/px**

a) The spectral information of the image sensor is vertically (Gaussian weighted) software binned by effectively 21.6 pixels for each spectral channel leading to 15.28 times pixel read noise.

6.) Supporting Figures

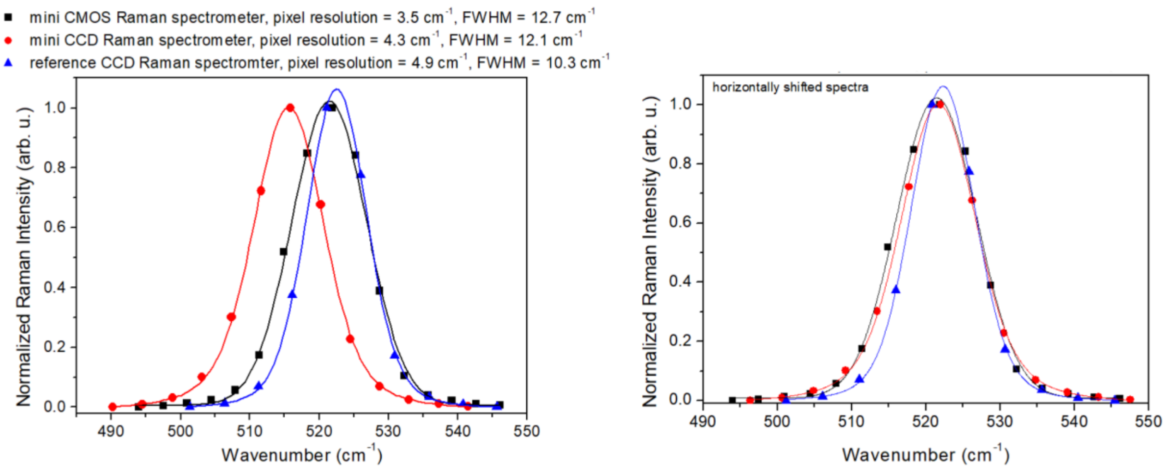

Figure S3. Raman marker of c-Si sample band fitted with a Voigt function: background corrected and normalized to unity. (A) original and (B) with corrected wavenumber axis.

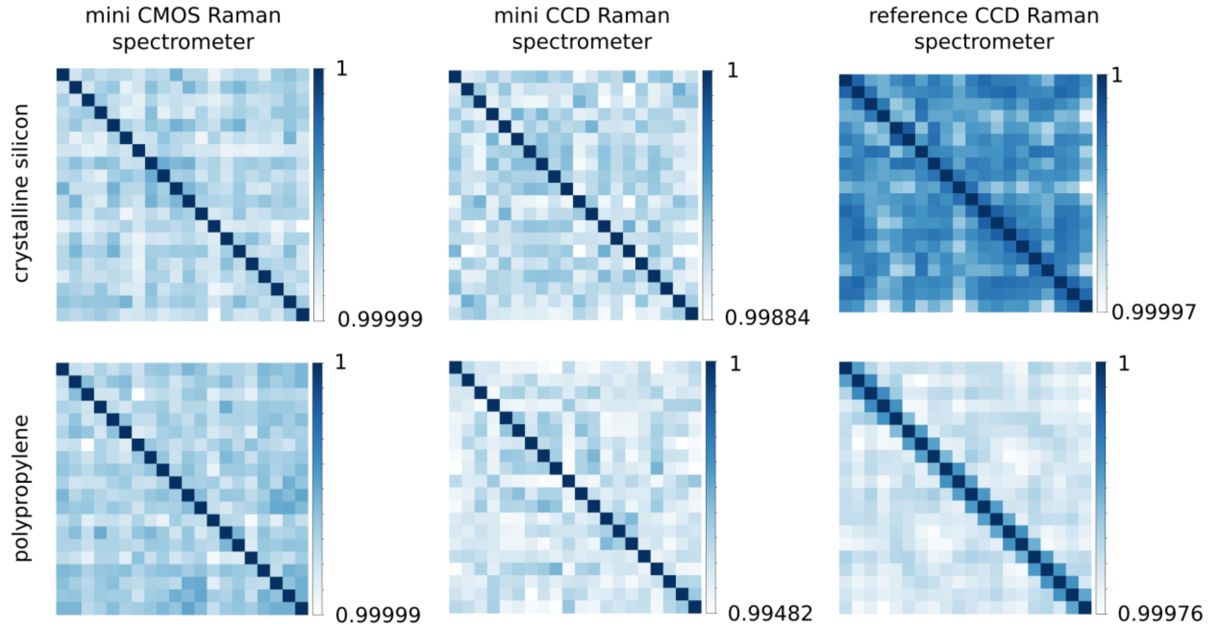

Figure S4. Map of correlation matrix. Each pixel of the map displays the correlation between two spectra. For better visualization just the first 20 spectra were here considered. Note the dark blue secondary diagonal in case of the reference CCD spectrometer indicating an averaging step during the readout of the CCD using Witec Control 4 software leading to improved SNR beyond shot noise limit.

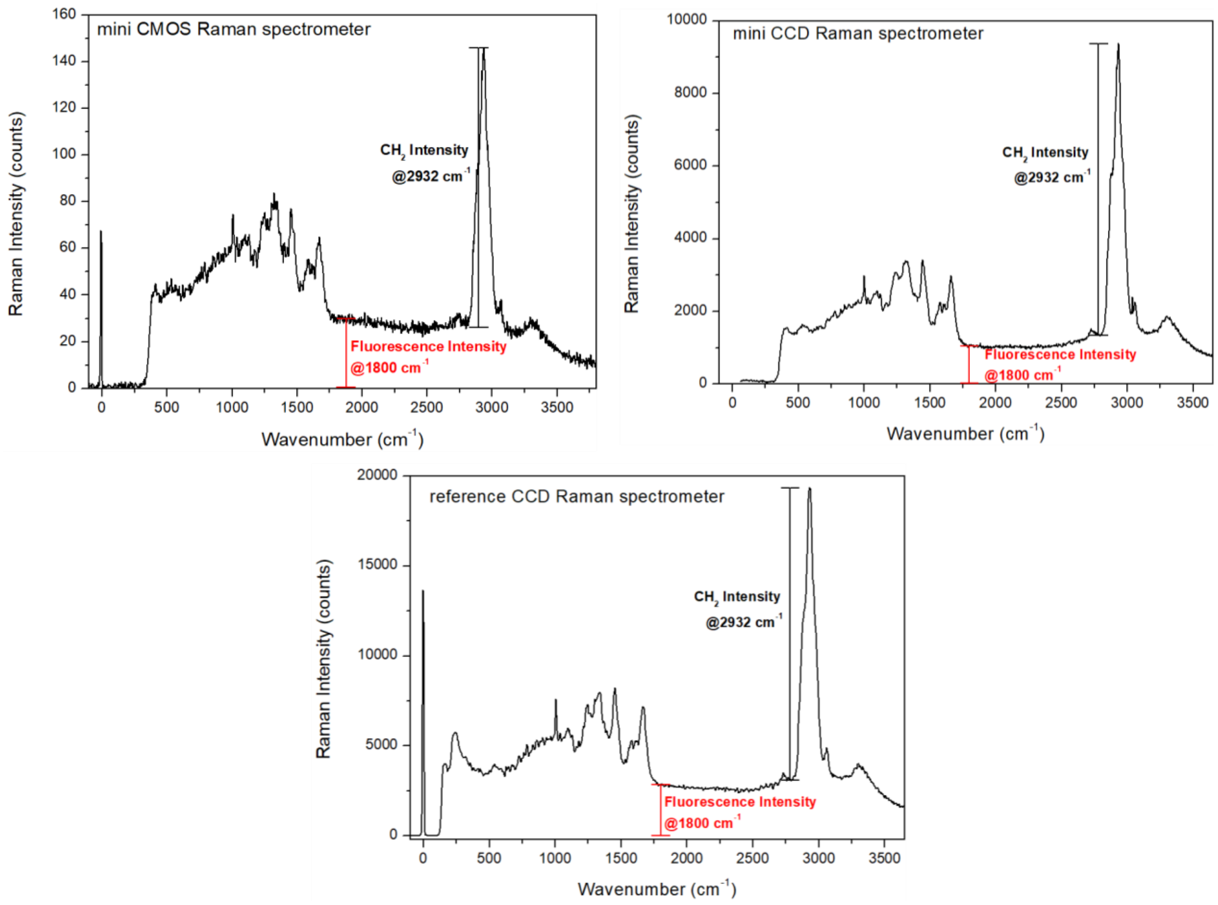

Figure S5. Dark corrected Raman spectrum of E. coli DSM 423 measured with the three Raman spectrometers (the last one of the measurement series at a selected position on the biofilm). The fluorescence intensity was measured in the silent region @1800 cm^{-1}; while the Raman band centered at 2932 cm^{-1} was chosen as Raman marker band.

References

1. McCreery, R. L., Signal-to-Noise in Raman Spectroscopy. In *Raman Spectroscopy for Chemical Analysis*, John Wiley & Sons, Inc: 2000; pp 49-71.
2. L.J. van Vliet; D. Sudar; Young, I. T., Digital Fluorescence Imaging Using Cooled CCD Array Cameras. In *Cell Biology*, Celis, J., Ed. Academic Press: New York, 1998; Vol. III, pp 109-120.
3. Aggarwal, R. L.; Farrar, L. W.; Saikin, S. K.; Aspuru-Guzik, A.; Stopa, M.; Polla, D. L., Measurement of the absolute Raman cross section of the optical phonon in silicon. *Solid State Communications* **2011,** 151, (7), 553-556.
4. Mertz, J., *Introduction to Optical Microscopy*. 2 ed.; Cambridge University Press: Cambridge, 2019.
5. FAK, C. Brechungsindex von Silicon. https://www.filmetrics.de/refractive-index-database/Si/Silicon
6. Wendel, H., Theoretical study of the Raman cross section and its pressure dependence in silicon. *Solid State Communications* **1979,** 31, (6), 423-426.
7. CMOSIS, CMV2000-datasheet-v3.9. In 2015; p 63.
8. Technology, A., iDus 401 Series. In 2012; pp 1-6.
9. Avantes, AvaSpec-ULS2048x64TEC-EVO SensLine Thermoelectrically Cooled Fiber-Optic Spectrometer. 1-2.
10. Toporski, J.; Dieing, T.; Hollricher, O., *Confocal Raman Microscopy*. 2 ed.; Springer, Cham: p XXIV, 596.
